# Sex, gender, and sexuality and the oral health workforce in Africa

**DOI:** 10.3389/froh.2025.1658158

**Published:** 2025-10-23

**Authors:** Moréniké Oluwátóyìn Foláyan, Kehinde Okanlawon, Adeyinka Ganiyat Ishola, Bridget Haire

**Affiliations:** 1 Department of Child Dental Health, Obafemi Awolowo University, Ile-Ife, Nigeria; 2 Social Inclusion, Justice and Empowerment Initiative, Minna, Nigeria; 3 Department of Nursing, University of Ibadan, Ibadan, Nigeria; 4 School of Public Health and Kirby Institute, University of NSW, Sydney, NSW, Australia

**Keywords:** gender equity, sexual and gender minorities, structural stigma, criminalization, health inequity, intersectionality, workforce homogeneity, research ethics

## Abstract

This study explores how sex, gender, and sexuality intersect with Nigeria's oral health workforce crisis, highlighting structural barriers that hinder inclusion and equitable care. Nigeria faces an acute shortage of dental professionals—just 0.03 dentists per 100,000 people—amid entrenched inequities shaped by colonial, patriarchal, and heteronormative systems. Originally designed to center the voices of sexual and gender minority (SGM) oral health professionals through interviews, the study encountered complete refusal to participate. This collective silence redirected the research focus, reframing non-participation as evidence of systemic stigma, legal risk, and fear of professional ostracization. Using the frameworks of structural stigma and intersectionality, the paper argues that SGM invisibility reflects institutionalized oppression that restricts workforce diversity and perpetuates health disparities. Conventional methods that emphasize “giving voice” are critiqued for failing to address power dynamics. In response, the study proposes alternative methodologies, including researcher positionality, autoethnography, and critical discourse analysis, to analyze silence as a form of resistance and evidence of structural erasure. The paper positions that a homogeneous workforce lacking cultural competence contributes to care avoidance and delayed treatment among SGM patients, who already face higher oral disease burdens. Addressing these inequities requires systemic interventions aligned with the WHO 2022 Oral Health Resolution's call for integrated, people-centered care, including decriminalization, inclusive workplace policies, gender-balanced leadership, SGM-affirming curricula, and rural workforce investment. The study concludes that oral health equity in Africa depends on transforming entrenched power structures to cultivate a diverse, inclusive, and responsive workforce that mirrors the identities and realities of the populations it serves.

## 1 Introduction

Oral health in Africa faces a dual challenge: a critically scarce workforce and systemic inequities rooted in gender, sexuality, and cultural norms. While the continent grapples with a shortage of dental professionals—only 0.03 dentists per 100,000 people in some regions (1, 2)—Nigeria's oral health sector reveals deeper layers of disparity. Recent studies highlight how sex, gender, and sexuality shape workforce dynamics and care outcomes (3), and the need to explore actionable strategies for building an inclusive, equitable oral health ecosystem in Africa.

The pursuit of equity in the oral health workforce is inextricably tied to understanding how sex, gender, and sexuality shape professional experiences and patient care outcomes. Yet, in many countries in Africa, systemic barriers and societal stigma render these dimensions of diversity invisible, silencing critical narratives. Our journey to explore these dynamics in Nigeria, a context where same-sex relationships are criminalized and sexual and gender minority individuals are heavily stigmatized (4), began with an attempt to amplify the voices of sexual minority individuals within the oral health workforce. We sought to conduct interviews with nine professionals known to the community as sexual and gender minority individuals, hoping to uncover their lived experiences of discrimination, resilience, and agency. However, not a single individual agreed to participate, nor would they complete a written qualitative interview guide. Their collective refusal became a critical data point, revealing a health system structured by fear, invisibility, and institutionalized marginalization. This silence, far from a methodological setback, became the cornerstone of our inquiry: *What does this collective refusal reveal about the structural and cultural forces governing sex, gender, and sexuality in Nigeria's oral health sector?*

This paper reframes the original intent of the study, shifting from a feature on individual narratives to an analysis of systemic erasure. Drawing on years of fieldwork observations, informal conversations with practitioners, and the sparse but revealing literature on sexual and gender minority individuals' invisibility in healthcare systems in countries in Africa, we argue that the reluctance to participate is not merely a research challenge but a symptom of institutionalized oppression. In contexts where sexual and gender minorities face criminalization and professional ostracization, silence becomes a survival strategy—one that perpetuates cycles of exclusion in the workforce and undermines equitable care for marginalized patients.

Theoretical frameworks of structural stigma (5) and intersectionality (6) anchor this analysis, illuminating how overlapping systems of power—patriarchy, homophobia, and professional hierarchies—constrain representation and reinforce health inequities. By situating Nigeria's oral health workforce within broader discourses on global health governance and human rights, this paper interrogates the ethical and practical dilemmas of researching marginalized populations in hostile environments. It also challenges conventional methodologies that prioritize “giving voice” to stigmatized groups without addressing the conditions that enforce their silence.

Ultimately, this work underscores a paradox: the absence of data on sexual and gender minorities in Nigeria's oral health workforce is itself a form of evidence. It reflects a system that renders certain identities unspeakable, with profound implications for workforce diversity, patient trust, and the global push for health equity. Through this lens, the paper contributes to urgent conversations about dismantling the architectures of exclusion that define too many health systems in countries in Africa.

## 2 Structural stigma and the ethics of silence

Structural stigma—the societal conditions, cultural norms, and institutional policies that systematically disadvantage marginalized groups—creates a formidable barrier to health workforce research and equity (5). While this analysis focuses on Nigeria, the dynamics of structural stigma are pervasive across much of Africa, varying in legal severity but consistently creating hostile environments for sexual and gender minority health professionals and researchers. In contexts like Nigeria, the Same-Sex Marriage (Prohibition) Act of 2013 not only prohibits marriage but also criminalizes the “public show of same-sex amorous relationship” and the registration or participation in “gay clubs, societies and organizations”. This creates tangible risks of arrest, professional ostracism, and violence (4, 7). This stigma manifests as a pervasive climate of fear, rendering sexual and gender minority individuals invisible within the oral health workforce. This invisibility is not a passive absence but an active, ethically significant phenomenon: silence functions as a strategy of survival, resistance, and critique against systems of structural violence.

The criminalization of SGM identities directly deters participation, with Nigeria's Same-Sex Marriage (Prohibition) Act (SSMPA) of 2013 serving as a stark example. The Act criminalizes not only marriage but also the “public show of same-sex amorous relationship”, creating tangible risks of arrest, professional ostracism, and violence (7). For health professionals, participating in research could be perceived as such a “show”, threatening their license and liberty. This legal hostility generates a profound chilling effect. Logie et al. (8) found that 45% of SGM individuals in Nigeria avoid healthcare settings due to anticipated discrimination, a reluctance that logically extends to research. This pattern is pan-African (9); in Uganda, the even more severe Anti-Homosexuality Act, 2023, has institutionalized fear, severely compromising public health outreach and research recruitment (10, 11). Hatzenbuehler's framework elucidates how such laws force marginalized groups to internalize fear, leading to the avoidance of institutions like research settings (5), a response compounded by the chronic stressors described in minority stress theory (12).

This dynamic is exacerbated by workplace discrimination, even in regions without explicit criminalization. Sexual and gender minority health professionals often face bias, harassment, and career limitations, fostering deep distrust in institutional initiatives (13–16). The fear of retaliation—such as being denied promotions or facing ostracization—discourages participation in studies, particularly those addressing diversity or discrimination. Intersectional identities further amplify these barriers. For instance, Black transgender women in Brazil face compounded discrimination due to racism and transphobia (17). In South Africa, which has constitutional protections, Black lesbian nurses report facing compounded discrimination, fostering deep distrust in institutional initiatives (18), illustrating how intersectional stigma systematically silences marginalized voices. Thus, even in the absence of criminalization, deep-seated stigma acts as a barrier. This illustrates that decriminalization, while necessary, is not sufficient.

These exclusions extend into research itself, where structural stigma shapes both design and ethics. Institutional Review Boards often impose stringent requirements for studies involving criminalized populations, delaying approvals or deterring sensitive inquiry (19). In Malaysia, where Sharia law criminalizes transgender identities, researchers face profound ethical dilemmas in balancing participant safety with data collection (20). Moreover, the absence of data disaggregated by gender identity or sexual orientation is a continent-wide problem and highlighted by the Lancet Commission on Oral Health (21). This conceals disparities and hinders evidence-based policymaking, especially for African contexts. The World Health Organization has underscored that inclusive health workforce data are critical to advancing Universal Health Coverage (22). Yet stigma continues to obstruct participation, silence marginalized groups, and perpetuate inequities. Addressing these barriers requires decriminalization, anti-discrimination policies, and intersectional methodologies that prioritize safety and trust (8). Researchers, policymakers, and institutions must confront these systemic obstacles, recognizing that dismantling structural stigma is not only a moral imperative but also a methodological necessity for equitable health workforce research.

This collective refusal to participate in research is far more than a methodological setback; it is a form of counter-conduct—a resistance to institutional power that historically extracts narratives without offering reciprocity or protection (23). It aligns with critiques of “helicopter research” and echoes Smith's decolonial argument that marginalized communities rightly distrust institutions that have weaponized knowledge against them (24). Silence, in this context, is not a lack of voice but a rejection of being commodified as “data points” within systems that deny their humanity (25). It exposes the limitations of traditional research ethics that prioritize “giving voice” without addressing the power imbalances that enforce silence, a point starkly framed by Spivak's question, “Can the subaltern speak?” (26).

The institutionalization of this silence has profound material consequences. Dental institutions, by failing to collect data on sexual and gender minority workforce experiences, reinforce the myth that these populations are non-existent or irrelevant (21). This erasure perpetuates a vicious cycle: the lack of sexual and gender minority representation in the professions fosters culturally incompetent care, which in turn leads sexual and gender minority patients to avoid clinics due to the anticipation of discrimination (27, 28). This avoidance exacerbates oral health disparities, as evidenced by the recent Nigerian data showing higher caries risk indicators among sexual minority adolescents and adults (29). Fakhrjahani et al.'s scoping review confirmed that LGBTQ+ populations globally face significant barriers to oral healthcare access and utilization, directly linking these barriers to experiences of stigma and discrimination within the system (30).

Confronting this reality demands a paradigm shift in both research and policy. Researchers must move beyond extractive methodologies, instead prioritizing safety, autonomy, and partnership with grassroots organizations (8). Ethically engaging with silence requires recognizing refusal not as an obstacle but as a critique of oppressive systems. Ultimately, dismantling this cycle necessitates systemic interventions: the decriminalization of sexual and gender minority identities (31), the implementation of anti-discrimination policies, and the reform of dental education and hiring practices to be truly inclusive (32). As hooks reminds us, “Speaking is a privileged act” (33); in contexts of structural stigma, silence is a wielded form of resistance. Centering this understanding is essential to transforming oral health systems into spaces where marginalized communities no longer need to refuse participation to protect themselves.

## 3 Workforce homogeneity and patient outcomes

The homogeneity of the healthcare workforce is a direct determinant of health equity. A lack of LGBTQ+ representation undermines cultural competence and perpetuates systemic biases, leading to tangible deficits in the quality of care for sexual and gender minority patients. This is not merely a diversity issue but a clinical one, as a non-diverse workforce fails to address the unique health needs of LGBTQ+ populations (34). Patients are more likely to disclose sensitive information and adhere to treatment when cared for by providers who share their identities or demonstrate cultural understanding (35). For instance, transgender patients report higher satisfaction when treated by providers knowledgeable about gender-affirming care (36).

Conversely, the absence of shared experience or specific training can result in critical gaps. Non-LGBTQ+ providers may lack awareness of specific health risks, such as elevated rates of mental health disorders (37, 38). This knowledge gap, compounded by implicit bias, often manifests as microaggressions or inadequate care, which fuels a well-documented cycle of avoidance (39, 40). Many LGBTQ+ adults avoid seeking care due to fear of discrimination, leading to delayed diagnoses of conditions like cancer and HIV (35, 41). This avoidance is exacerbated when patients perceive clinics without diverse staff as unwelcoming (42). Furthermore, systemic bias is evident in clinical decisions, such as the under-prescription of PrEP to populations at risk for HIV (43).

Therefore, diversifying the workforce is a pragmatic necessity. Targeted recruitment of LGBTQ+ students, supported by scholarships and mentorship, is crucial for building a representative pipeline (44, 45). Ultimately, a diverse workforce fosters peer accountability, promotes equitable practices, and is essential for building trust and improving health outcomes for marginalized populations (6, 46).

## 4 Alternative methodologies for research with sexual and gender minority individuals

When primary data collection is rendered inaccessible, whether due to systemic stigma, criminalization, or participant reluctance, researchers must pivot toward methodologies that center reflexivity, critique, and lived experience. In contexts like Nigeria, where same-sex relationships are criminalized and sexual and gender minority individuals’ identities are policed, traditional qualitative methods such as interviews often fail to capture marginalized narratives. This methodological impasse, however, invites innovative approaches that interrogate power structures while honoring the ethical complexities of researching oppressed populations. Three alternative strategies—researcher positionality, autoethnography, and critical discourse analysis—can transform silence into evidence and refusal into critique.

The first strategy is to employ the reflexive practice of researcher positionality—the explicit acknowledgment of how one's identity, power, and social location shape the research process. This can become a critical tool when primary data is inaccessible. In settings where marginalized groups fear retaliation, the researcher's own experiences and observations can illuminate systemic inequities. For instance, our inability to recruit sexual and gender minority oral health professionals in Nigeria reflects not a failure of methodology but a manifestation of structural stigma. By critically reflecting on our role as an insider-outsider (a researcher familiar with Nigeria's oral health sector but not part of its sexual and gender minority community, two researchers familiar with the sexual and gender minority community but not part Nigeria's oral health sector, and one researcher in neither of the communities), we can contextualize participants' silence as resistance to a hostile system.

Bourke (47) argues that positionality challenges the myth of researcher neutrality, urging scholars to “write themselves into the text” to expose power imbalances. In this case, our fieldwork observations, such as witnessing colleagues avoid discussions of sexuality in clinical training, reveal how heteronormativity is institutionalized in Nigerian dental education. These reflections, grounded in critical race and feminist theories (6), underscore how systemic erasure perpetuates workforce homogeneity and care disparities.

Second, the methodology of autoethnography bridges the personal and political, using the researcher's lived experiences to critique cultural norms (48). In the absence of participant voices, this method offers a pathway to document how oppression operates within the oral health workforce. For example, our informal conversations with dental professionals in Nigeria, where homophobic remarks were casually dismissed as “cultural norms”, serve as autoethnographic data. These encounters, analyzed through the lens of queer theory (49), expose how institutionalized homophobia silences sexual and gender minority professionals and normalizes exclusion.

Critics argue that autoethnography risks solipsism (50), but its value lies in its ability to humanize structural violence. By detailing my frustrations and ethical dilemmas during fieldwork, such as grappling with the complicity of academic institutions in sexual and gender minority individuals, I amplify the emotional toll of systemic oppression. This approach aligns with Smith's decolonial framework, which prioritizes Indigenous methodologies that resist extractive research (24). In Nigeria, autoethnography becomes an act of solidarity, centering marginalized perspectives without exploiting vulnerable participants.

Third, when marginalized voices are suppressed, critical discourse analysis deconstructs the language of power embedded in policies, media, and institutional rhetoric (51). Applying critical discourse analysis to Nigeria's oral health policies reveals glaring omissions: sexual and gender minority individuals' health is absent from national guidelines, and gender is reduced to a binary category. Similarly, analyzing media portrayals of sexual and gender minority individuals as “immoral” or “un-African” (52) exposes how public discourse legitimizes discrimination in healthcare settings.

Critical discourse analysis also interrogates silences. The refusal of dental schools in Nigeria to address sexuality in curricula, despite high rates of HIV among men who have sex with men in Nigeria (53), reflects a discursive strategy to maintain patriarchal heteronormative control. By juxtaposing these omissions against global health mandates like the World Health Organization's call for inclusive care, critical discourse analysis highlights the contradictions between Nigeria's commitments to universal health coverage and its exclusionary practices. This method, rooted in Foucault's theories of biopower (23), underscores how language constructs realities that marginalize.

Combining these strategies creates a robust methodology for analyzing inaccessible phenomena. Researcher positionality grounds the study in ethical reflexivity, autoethnography personalizes systemic critique, and critical discourse analysis dismantles oppressive discourses. Together, they challenge the notion that “no data” equates to “no problem”—instead, they reveal how silence is produced and weaponized. In Nigeria's oral health sector, a reflection of what happens in many countries in Africa, this triangulation exposes the role of colonial-era laws (e.g., Section 214 of the Nigerian Criminal Code) in legitimising sexual and gender minority individuals’ exclusion, the complicity of dental institutions in perpetuating gender-based violence through curricular neglect, and the global north-south power imbalances that prioritise Western narratives of sexual and gender minority individuals’ rights and emphasis on “coming out” over localised resistance. Furthermore, the narrow and exclusionary definition of oral health overlooks and marginalizes the unique experiences of sexual and gender minority individuals, contributing to their systemic invisibility within oral health discourse and practice (54).

These methodologies demand a redefinition of rigor. Rather than privileging participant voices, it centers the researcher's responsibility to critique systems that enforce silence. This aligns with Smith's decolonial imperative to “research back” against oppressive structures (25). Moreover, they challenge Eurocentric methodologies that often disregard the geopolitical realities of countries in Africa, where researchers navigate state surveillance and communal stigma. In contexts where primary data is inaccessible, alternative methodologies are not mere substitutes but radical acts of resistance. By leveraging positionality, autoethnography, and critical discourse analysis, researchers can transform absence into evidence, illuminating how power operates in silence. For Nigeria's oral health workforce, this approach helps to document inequities and imagine futures where marginalized professionals and patients are no longer rendered invisible. This methodological shift redefines rigor as the courage to confront silence, offering a blueprint for equity-focused research in hostile environments.

## 5 The intersectional crisis: workforce scarcity meets systemic bias

Africa's oral health workforce crisis is defined by systemic inequity, where scarcity collides with the intersecting forces of gender discrimination and state-sanctioned homophobia. This intersectional crisis is epitomized by the experiences of women who identify as sexual minorities, a demographic rendered invisible in research yet disproportionately impacted by patriarchal norms and structural stigma (55).

Even as more women enter dental schools (56), gender inequality persists. Women in Nigeria's oral health sector remain underrepresented in leadership and academia, facing a “glass ceiling” despite often outperforming male counterparts in research productivity (57, 58). For sexual minority women, these professional barriers are magnified exponentially. Laws criminalizing same-sex relationships force the concealment of identity as a survival strategy, creating workplaces fraught with fear and professional ostracism. This results in a twofold exclusion: they are hypervisible targets of discrimination yet entirely invisible in policy and research agendas.

This erasure has profound consequences for the entire health system. The failure of the oral health workforce to reflect the diversity of the populations it serves—in terms of gender, sexuality, and geography—directly undermines trust and accessibility for the most vulnerable communities. The complete absence of studies examining how gender and sexual minority identities shape professional participation in African dentistry is not an accident but a symptom of systems that equate research with risk. Dismantling these barriers requires confronting the legacies of colonialism and patriarchy through policies that decriminalize same-sex relationships and mandate equity. Until then, the workforce will remain a mirror of its inequities—scarce, fragmented, and exclusionary by design.

## 6 Multidisciplinary solutions for equity

Achieving equity in the oral health workforce demands more than isolated interventions requires a multidisciplinary, systemic overhaul that confronts the intersecting barriers of gender, sexuality, and geography. Central to this transformation is the intentional diversification of the workforce itself. For workforce planning and recruitment, targeted recruitment of individuals from underrepresented genders and sexual minorities is a foundational strategy to dismantle homogeneity. This effort must be coupled with decriminalization and robust anti-discrimination policies, which are not merely human rights imperatives but essential retention tools. Without these legal protections, recruitment efforts are undermined, as professionals from marginalized groups cannot safely practice, particularly in rural areas where their presence is most needed to build community trust (1, 59). This effort is bolstered by the WHO's 2022 Oral Health Resolution (WHA74.5) (60), which urges member states to integrate oral health into universal health coverage and address social determinants, providing a critical policy lever to mandate inclusive, non-discriminatory care standards.

For dental education and accreditation, education reform serves as the critical bridge between policy and practice. Integrating inclusive curricula on cultural humility, trauma-informed care, and sexual and gender minority health is necessary to achieve competency-based training goals. For example, modules on gender-based violence equip providers to recognize oral injuries linked to intimate partner violence (61), while training on managing hormone therapy-related xerostomia in transgender patients addresses a specific clinical need (62). These reforms must be validated through accreditation standards that prioritize equity, as outlined by bodies like the American Dental Education Association (30), ensuring graduates are prepared to serve all populations.

For clinical service delivery, these educational and workforce changes directly impact the quality of care. A diverse, well-trained workforce is fundamental to delivering trauma-informed care that fosters patient disclosure and adherence. By competently addressing the oral health implications of issues like HIV or hormone therapy (63), providers can move beyond a narrow clinical focus to offer holistic, affirming clinical service delivery that reduces care avoidance and improves outcomes for sexual and gender minority patients.

Ultimately, these solutions are interdependent. Workforce diversification falters without policies that protect marginalized professionals; policy gains remain theoretical without educators to operationalize them. By uniting advocates, educators, and policymakers across sectors, the oral health field can transcend its legacy of exclusion and become a beacon of equity, one where every patient and professional, regardless of gender or sexuality, thrives. There are ongoing shifts in gender perspectives through the confrontation of patriarchal standards in Africa influenced by globalization, activism, and the inclusion of gender education into the academic curriculum (64). Efforts to diversify the oral health workforce in Africa can build on this momentum.

## 7 Call to action: From reflection to courageous praxis

Our internal dialogue, grounded in an acknowledgment of our own privileges and marginalizations, revealed that the greatest barrier to equity is often not a lack of evidence, but a surplus of fear. This fear, rooted in structural stigma, silences advocacy and perpetuates the very inequities our research outlines. Therefore, our call to action is not merely for policy change but for a fundamental shift in scholarly and professional courage, guided by the principles of Freirean praxis and bell hooks' engaged pedagogy (65, 66).

We challenge academic and health institutions to actively create “safe enough” spaces for dissent—forums where power dynamics, gender, and sexuality can be discussed without fear of reprisal. This is the essential precursor to tangible change. In addition, professional associations must use their influence to mandate cultural humility and SGM-affirming clinical competencies as a requirement for accreditation and continuing education, transforming pedagogical norms.

For policymakers and health administrators, the mandate is clear: operationalize intersectionality in line with global commitments like the WHO Oral Health Resolution, which frames equity as a cornerstone of effective health systems (60). This requires enacting and enforcing workforce development policies that include targeted scholarships and mentorship programs for marginalized students. It demands the explicit inclusion of oral health in decriminalization efforts and the establishment of clear, accountable standards for inclusive care within public health systems. Investment must also be directed toward rural workforce initiatives that address geographic inequity. These are not aspirational goals but practical, achievable steps toward a workforce that reflects Africa's dynamic diversity.

Furthermore, researchers must collectively abandon extractive methodologies. In partnership with communities, adopt participatory, arts-based approaches like photovoice (67) that cede narrative power and ensure ethical engagement. The primary ethical duty is to the safety and agency of participants, not to data extraction.

The confession of a co-author, advised to remain silent for career safety, is a microcosm of the systemic problem. Thus, our primary call is for reflections on vulnerability not as a weakness, but as a radical, justice-oriented tool. It underscored that speaking out, particularly in hostile or risk-laden environments, is both an act of defiance and a necessary step toward transformation. Africa's oral health crisis may also reflect a crisis of exclusion. Workforce scarcity collides with gendered, sexual, and geographic inequities, rendering marginalized communities invisible. Our call is not theoretical: it demands that each sector—education, policy, and research—fulfill its specific role in dismantling structural stigma through decolonial research, inclusive policy, and the courage to center silenced voices. As Smith reminds us, justice is seized through relentless praxis (25) —not given. Let this be our starting point.

## 8 Conclusion

The path to resolving Africa's oral health crisis runs directly through the dismantling of its systemic biases. A response that addresses only workforce scarcity without confronting the intersecting crises of gender discrimination and structural homophobia will have limited success. The evidence is clear: equity is not a secondary concern but the foundational principle upon which an effective, trustworthy health system must be built. True progress, therefore, hinges on our collective willingness to engage in the relentless work of praxis—where critical reflection on privilege and power fuels tangible action. It demands that we reimagine our institutions not as sites of control, but as ecosystems of liberation where every voice, especially those historically silenced, shapes the future of care. Justice for Africa's marginalized populations will not be granted by existing systems; it must be seized through courageous, collaborative effort (22). Let this synthesis of analysis, reflection, and actionable strategy be our starting point.

## Data Availability

The original contributions presented in the study are included in the article/Supplementary Material, further inquiries can be directed to the corresponding author.
